# Prevalence of subclinical keratoconus and impact on adults undergoing routine, uncomplicated age-related cataract extraction

**DOI:** 10.3389/fopht.2023.1269439

**Published:** 2023-09-20

**Authors:** Tu M. Tran, Aman Mittal, Vihar Naik, Priyanka Chhadva, Matthew Wade, Sumit Garg

**Affiliations:** ^1^ Department of Ophthalmology and Visual Neurosciences, University of Minnesota, Minneapolis, MN, United States; ^2^ Dean McGee Eye Institute, University of Oklahoma, Oklahoma, OK, United States; ^3^ School of Medicine, University of California, Irvine, Irvine, CA, United States; ^4^ Gavin Herbert Eye Institute, University of California, Irvine, Irvine, CA, United States

**Keywords:** cataract, keratoconus, prevalence, subclinical keratoconus, tomography, topography

## Abstract

**Aim:**

To determine the prevalence of subclinical keratoconus (SKCN) among individuals undergoing routine, uncomplicated age-related cataract surgery and its impact on visual and refractive outcomes.

**Patient and Methods:**

At a major academic ophthalmology department in the United States, we reviewed records of patients aged 50 years and older who underwent surgery from January 2011 to June 2022. We excluded patients who had poor-quality or unreliable tomographic data, previous corneal surgery, keratorefractive procedures, and significant vision-limiting ocular pathology. We defined SKCN if an eye had a Belin-Ambrósio enhanced ectasia index (BAD-D) ≥1.7, which was based on the results of a meta-analysis of large studies. In addition to the BAD-D cutoff, the eye had to deviate significantly on at least one of seven additional parameters: 1) posterior elevation at thinnest point, 2) index of vertical asymmetry, 3) index of surface variation, 4) total front higher order aberrations, 5) front vertical coma, 6) front secondary vertical coma, 7) back vertical coma. An individual had SKCN if at least one eye met the tomography-based classification and did not have manifest KCN in either eye. Visual and refractive outcomes data were acquired from patients of one experienced cataract surgeon with cases done from July 2021 to June 2022. Statistical significance was set at p < 0.05.

**Results:**

Among 5592 eyes from 3828 individuals, the prevalence of SKCN was 24.7% (95% CI, 23.4 – 26.1, 945 individuals), and the prevalence of KCN was 1.9% (95% CI, 1.6 – 2.4, 87 individuals). The prevalence of SKCN did not increase with age and was more prevalent among females and non-white races. Median post-operative month one distance-corrected visual acuity (DCVA) and proportion of eyes with improvement in DCVA were similar between normal and SKCN eyes. The proportion of eyes reaching ±0.5 and ±1.0 diopter within the refractive target were similar between normal and SKCN eyes.

**Conclusion:**

SKCN is highly prevalent and should be detected but is unlikely to have a significant deleterious effect on outcomes in routine, uncomplicated cataract surgery.

## 1 Introduction

Keratoconus (KCN) is a progressive, typically bilateral corneal ectasia in which the cornea progressively thins and steepens, resulting in significant vision loss in advanced stages. The most recent global estimate of the prevalence of KCN is 0.14% based on a meta-analysis (1), in which some of the sourced studies reported a prevalence as high as 8.9% (2). Advancements in corneal cross-linking have enabled safe and early stabilization with good long-term prognosis (3, 4). Thus, there has been a major impetus for earlier KCN detection, also catalyzed, in part, by efforts to screen out at-risk corneas from undergoing laser vision correction (5). This general paradigm shift toward earlier diagnosis has led to further recognition of subclinical keratoconus (SKCN).

SKCN necessarily lacks clinical signs, significant visual impairment, and large deviations from normative tomographic parameters. The earliest ectatic changes occur in the posterior cornea (6). Improvements in Scheimpflug tomographic imaging have enabled accurate modeling and estimation of the posterior corneal surface, and the development of more robust data acquisition methods have made detection of SKCN more practical at scale (7–9). The knowledgebase gained from the keratorefractive and KCN tomographic literature has, however, yet to be applied to the population undergoing age-related cataract extraction. Among those age 65 years and older in the United States, the prevalence of KCN was reported at 0.0185% in 2003 (10), though SKCN was not diagnosed by tomographic imaging at the time. Clinical experience from our setting suggests the prevalence of SKCN among aging adults is substantial, though this has yet to be quantified. We surmise that knowing the disease burden might alter practice patterns, such as routine use of tomographic imaging in pre-operative evaluation, more appropriate intraocular lens (IOL) selection, and better prognostication (setting expectations for post-operative visual outcomes). We therefore undertook this study to describe the basic epidemiology of SKCN and its impact on visual and refractive outcomes in adults undergoing routine age-related cataract extraction.

## 2 Patients and methods

This was a retrospective analysis of adults aged 50 years and older who underwent cataract extraction and IOL implantation at a tertiary-referral academic eye center in the United States, between January 2011 and June 2022. Exclusion criteria were previous or concurrent corneal surgery (e.g., all kinds of keratoplasty including Descemet’s membrane endothelial keratoplasty, keratorefractive surgery, pterygium and ocular surface lesions excision surgery) and concomitant pathology limiting visual acuity potential, such as significant corneal disease (e.g., scar, Salzmann), retinal pathology, advanced-stage glaucoma, visually significant optic neuropathies, and low-quality or unreliable tomographic data (e.g., due to severe ocular surface disease or contact-lens warpage). Secondary IOL procedures were excluded. Patients with mild to moderate-stage glaucoma undergoing combined cataract surgery and minimally invasive glaucoma surgery (MIGS) were included. This study was approved by the institutional review board of the University of California, Irvine (UCI, reference # HS 2020-6160).

The study aimed for sample size of 1475 individuals based on n = Z_α/2_^2^p(1-p)/L^2^, where Z_α/2 = _1.96, p = 0.04 prevalence proportion of KCN at a population level (2), and L^2^ is accepted margin of error of 0.01. We justify 4.0% since the only study reporting SKCN prevalence at a population level showed a 4.4% prevalence (2). We assume that based on high accessibility of the United States’ adult population, that almost all adults who need cataract surgery would eventually undergo surgery; hence, the 4.0% assumption for a clinic-based setting can closely approximate the underlying base population. Secondly, a lower 4.0% estimation is more stringent and requires a larger sample size than 4.4%.

### 2.1 Clinical data

For each individual, we extracted from the medical record the data on general demographics, medical co-morbidities, and ocular co-morbidities including KCN and SKCN. For a subset of patients operated on by one senior cataract surgeon (SG) during a one-year period, data were extracted on distance corrected visual acuity (DCVA) at baseline evaluation and at post-operative month one, manifest refraction at baseline evaluation and at post-operative month one, target refraction, and intraoperative details. During the study period, all cataract extractions were performed using phacoemulsification with a subset of patients undergoing femtosecond laser-assisted capsulorrhexis, nuclear fragmentation, and astigmatic incisions.

### 2.2 Corneal tomography

Patients underwent pre-operative tomography using a Scheimpflug camera (Pentacam HR, OCULUS Optikgeräte GmbH, Wetzlar, Germany). Artificial tear solution was used in those with evidence of dry eye syndrome (DES), and individuals with severe DES underwent further intense therapy, including a short course of topical corticosteroids, prior to final pre-operative biometric calculations and tomography. Soft contact lens and rigid gas permeable contact lens wearers were asked to avoid lens use for at least one week to one month or more prior to tomography. Data from the most proximal pre-operative tomograms were used in this study. Data were extracted using Oculus’ built-in export program.

### 2.3 Keratoconus and subclinical keratoconus definitions

KCN was diagnosed clinically (e.g., asymmetric refraction with high astigmatism, anterior segment examination findings) with supplementation by tomographic data, including classical KISA index constituents [e.g. central keratometry >47.2D, inferior-superior dioptric asymmetry >1.4D, skewed radial axis >21°, and corneal astigmatism index (SimK_1_-SimK_2_) >1.5D] (11).

**Figure 1:**
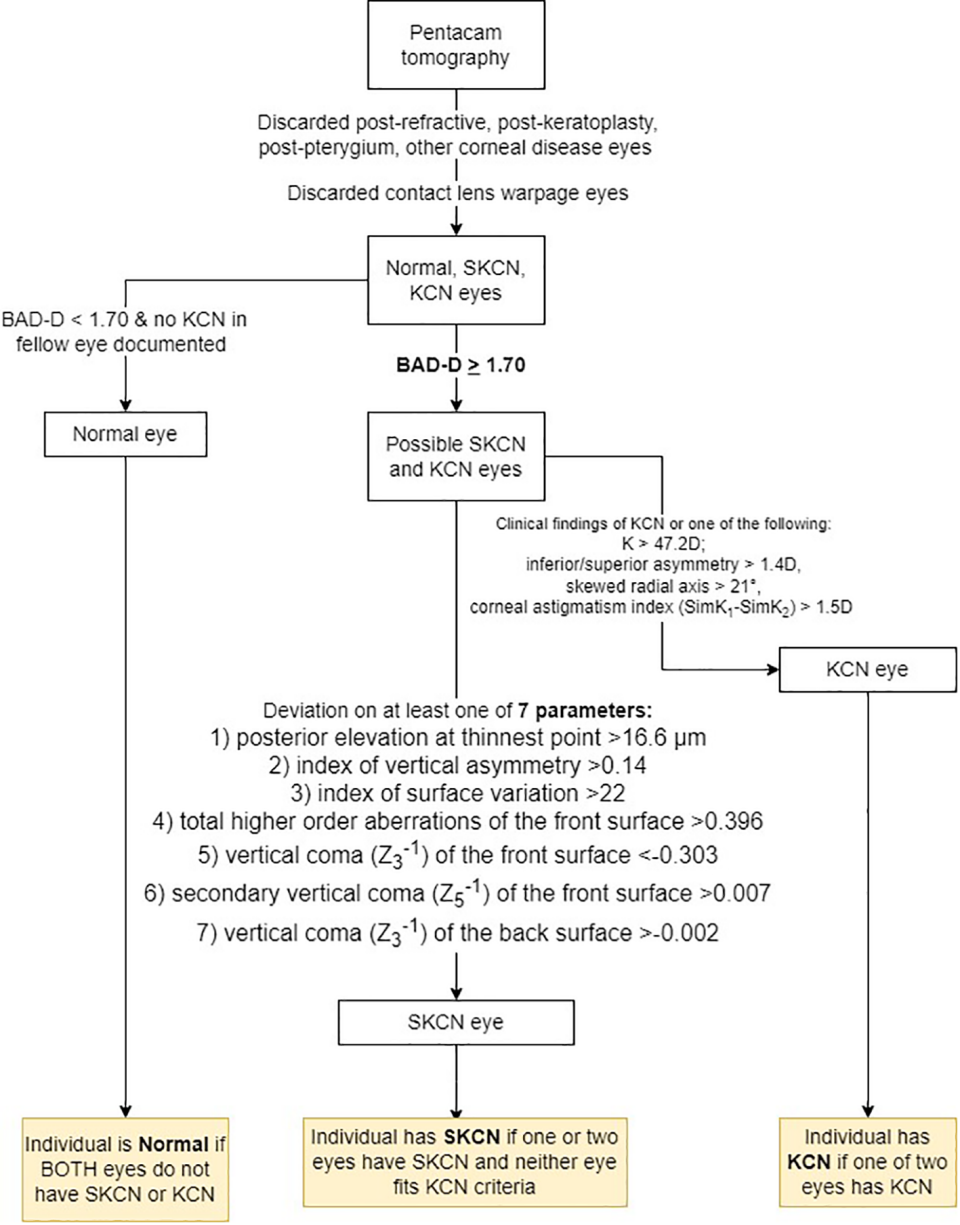
Process for classifying eyes as normal, subclinical keratoconus (SKCN) or keratoconus (KCN). BAD-D, Belin/Ambrósio final index.

There is no universally accepted diagnostic criteria of SKCN in the literature, though several studies have established cutoffs for tomographic parameters indicative of SKCN by assessing the fellow eye of one with manifest KCN (12). For eyes that were not already classified as KCN, we first screened these eyes with the BAD-D, then assessed if one of seven other variables were outliers. First, the BAD-D was the major screen-in variable using a cutoff ≥1.70. This cutoff was determined using a mixed-effects, maximum-likelihood model to generate a pooled estimate of the mean (theta) and standard deviation (tau) of BAD-D among normal eyes. The upper limit of the 99.9% confidence interval for this metanalysis was 1.697 (**Table 1**). This pooled estimate was based on seven robust studies reporting mean BAD-D and SD using validated methodology described by Wan and colleagues (19). The ages of patients in these studies were similar, ranging mid-twenties to mid-thirties, thus even though the BAD-D was computed using age-matched controls from the Pentacam normative database, the 1.7 cutoff is still valid for more elderly patients since it represents 1.7 standard deviations above the mean for age-matched normal eyes. It is important to note this 1.7 cutoff exceeds the manufacturer’s suggested cutoff of 1.6. After the BAD-D criterion was met, to be considered as SKCN the eye must exhibit deviation in at least one of seven additional parameters: 1) posterior elevation at thinnest point ≥16.6 μm, 2) index of vertical asymmetry (IVA) ≥0.14, 3) index of surface variation (ISV) ≥22, 4) total higher order aberrations (HOA) of the front surface >0.396, 5) vertical coma (Z_3_^-1^) of the front surface <-0.303, 6) secondary vertical coma (Z_5_^-1^) of the front surface >0.007, and 7) vertical coma (Z_3_^-1^) of the back surface >-0.002. These cutoffs have been determined by previous well-designed tomographic studies on SKCN in young adult populations (5, 20, 21). In summary, provided that the eye did not demonstrate any clinical features of manifest KCN, did not have exceedingly abnormal tomographic values in the KCN range, did not have existing diagnosis of KCN in the medical record, and met the two-stage tomographic-based screening process described above, then the analyzed eye was classified as SKCN. Regarding the prevalence proportion calculation, the individual was counted as SKCN if the individual had two SKCN eyes, or one SKCN and one normal. Any individual with one KCN eye was counted as KCN. This process is summarized in **Figure 1**.

**Table 1:** Studies used in pooled estimate of Belin-Ambrósio enhanced ectasia index (BAD-D).

Study name	N	Age mean control	n control	Mean control	SD control	n SKCN	Mean SKCN	SD SKCN	n KCN	Mean KCN	SD KCN
Steinberg et al., 2015 (13)	635	33	196	1.3	1.3	146	2.4	1.8	293	11.2	7.8
Muftuoglu et al., 2015 (14)	224	29	134	0.57	0.59	45	1.49	0.82	45	6.49	3.22
Hashemi et al., 2016 (15)	647	29.6	200	0.96	0.8	63	3.34	2.9	384	9.55	5.35
Luz et al., 2016 (16)	97	25.7	76	0.52	0.5	21	1.84	1.34	0.52		
Awad et al., 2017 (17)	240	26.6	144	1.29	0.6	48	1.4	0.5	48	6.7	2.5
Koc et al., 2020 (18)	602	26	300	0.96	0.58	151	2.05	0.87	151	7.29	3.44
Pooled estimates				0.841 (99.9%CI -0.015, 1.697)			1.628 (99.9%CI 0.449, 2.807)			7.218 (99.9%CI 1.964, 12.471)	

CI, confidence interval; SKCN, subclinical keratoconus; KCN, manifest keratoconus.

Mean and standard deviation (SD) are of the BAD-D value for controls, SKCN, and KCN.

### 2.4 Statistical analysis

We used descriptive statistics and frequency analysis on all continuous and categorical variables among demographics, clinical and surgical data, and tomographic data. We calculated prevalence using Taylor series linearization as a bootstrapping method, and we analyzed each eye as the ultimate unit of analysis, while the individual was used to link two eyes in bilateral cases. This is a widely accepted method for variance calculation in large population studies (Demographic and Health Surveys, www.dhsprogram.com). We applied appropriate significance testing for univariate associations, such as t-test and linear regression for continuous parametric data, proportion test for dichotomous data, Wilcoxon rank-sum test for non-parametric, or logistic regression for binary data. We set statistical significance to p < 0.05. We performed statistical analyses with STATA 17 (StataCorp, College Station, Texas, United States), and we managed the database with Microsoft Excel (Microsoft Corp, Redmond, Washington, Untied States).

## 3 Results

### 3.1 Demographics, ophthalmic and medical characteristics

There were 16,951 eyes available for inclusion. After excluding eyes lacking high quality, reliable tomographic data (excluding 7491 eyes) and applying the exclusion criteria described in the methods section (excluding 3868 eyes), there were a total of 5592 eyes from 3828 individuals included for analysis (32.9% of all available eyes). Demographics, ocular, and medical history categorized by normal, SKCN, and KCN are provided in **Table 2**. Comparing the normal and SKCN groups, there was no significant difference between age at time of surgery (mean 72.0 versus 72.7 years), levels of body mass index, smoking history, or co-morbid, possibly confounding ocular conditions such as DES and blepharitis. There were more females in the SKCN group (59.7% versus 54.1%) and more non-white individuals in the SKCN group (40.7% versus 24.8%). Diabetes mellitus type 2 and primary hypertension were more prevalent among those with SKCN. Atopy was more prevalent among the normal group, though this was not statistically significant.

**Table 2:** Patient general demographic and medical characteristics (N = 3828 individuals).

	Normal (n = 2796)	SKCN (n = 945)	KCN (n = 87)	Difference between normal and SKCN (*P* value)
Age at surgery: mean ± standard deviation (range)	72.0 + 8.3 (50.1 – 97.3)	72.7 ± 8.2 (50.4 – 96.2)	70.3 ± 9.1 (50.1 – 88.8)	0.0544
Female, n (%)	1514 (54.1)	564 (59.7)	42 (48.3)	0.003
Race/ethnicity, n (%)				
White	2103 (75.2)	561 (59.3)	45 (51.4)	<0.001
Hispanic	254 (9.1)	104 (11.0)	12 (13.5)	
Asian	150 (5.4)	166 (17.6)	14 (16.2)	
Other/Mixed race	220 (7.9)	83 (8.8)	14 (16.2)	
Black	69 (2.5)	31 (3.3)	2 (2.7)	
Body mass index [BMI] (kg/m^2^)				
Underweight (< 18.5)	94 (3.4)	33 (3.5)	3 (3.7)	0.073
Normal (18.5 – 24.9)	1162 (41.5)	415 (43.9)	35 (40.7)	
Overweight (25 – 29.9)	1050 (37.5)	348 (36.8)	23 (25.9)	
Obese (30 – 39.9)	451 (16.1)	149 (15.8)	24 (27.8)	
Morbidly obese (≥ 40)	39 (1.4)	0 (0.0)	2 (1.9)	
Smoking status, n (%)				
Never smoked	1954 (69.9)	682 (72.1)	79 (90.7)	0.376
Ex-smoker	763 (27.3)	238 (25.1)	6 (7.4)	
Occasional smoker	28 (1.0)	3 (0.4)	0 (0.0)	
Daily smoker	51 (1.8)	22 (2.4)	2 (1.9)	
Allergic conjunctivitis, n (%, 95%CI)	347 (12.4, 11.2-13.7)	124 (13.1, 10.6-14.4)	11 (12.5, 7.8-16.3)	0.361
Blepharitis, n (%, 95%CI)	570 (20.4, 18.4-22.5)	174 (18.4, 15.4-21.9)	18 (20.4, 11.5-33.5)	0.313
Dry eye syndrome, n (%, 95%CI)	1625 (58.1, 55.6-60.6)	540 (57.2, 53.0-61.3)	53 (61.1, 47.3-73.3)	0.672
Asthma history, n (%, 95%CI)	231 (8.3, 7.0-9.7)	79 (8.4, 6.3-11.0)	6 (7.4, 2.7-18.5)	0.913
Atopic dermatitis, n (%, 95%CI)	47 (1.7, 1.1-2.4)	9 (0.91, 0.38-2.2)	2 (1.8, 0.2-12.5)	0.197
Atopy, n (%, 95%CI)	238 (8.5, 7.2-10.0)	55 (5.8, 4.1-8.1)	2 (1.8, 0.2-12.5)	0.061
Diabetes mellitus type 1, n (%, 95%CI)	51 (1.8, 1.2-2.6)	14 (1.4, 0.73-2.9)	0 (0.0)	0.649
Diabetes mellitus type 2, n (%, 95%CI)	554 (19.8, 17.9-21.9)	272 (28.8, 25.1-32.7)	23 (25.9, 15.8-39.5)	<0.001
Hypertension, primary, n (%, 95%CI)	1351 (48.3, 45.8-50.8)	511 (54.1, 49.9-58.2)	44 (50.6, 36.7-63.3)	0.021
Major depressive disorder, n (%, 95%CI)	314 (11.2, 9.7-12.9)	103 (10.9, 8.6-13.8)	3 (3.7, 0.89-14.02)	0.977
Migraine, n (%, 95%CI)	146 (5.2, 4.2-6.4)	48 (5.1, 3.5-7.3)	0 (0.0)	0.963
Obstructive sleep apnea, n (%, 95%CI)	355 (12.7, 11.1-14.5)	114 (12.0, 9.6-15.02)	10 (11.1, 5.0-22.9)	0.698
Rheumatic and connective tissue diseases, n (%, 95%CI)	98 (3.5, 3.3-3.7)	34 (3.6, 3.2-4.0)	3 (3.1, 2.03-4.5)	0.662

### 3.2 SKCN epidemiology

The overall prevalence of SKCN was 24.7% (95% CI, 23.4 – 26.1, 945 of 3828 individuals). Individuals with KCN in at least one eye were classified as KCN; the overall prevalence of KCN in our cohort was 1.9% (95% CI, 1.6 – 2.4, 87 of 3828 individuals). The remainder had unremarkable clinical examinations regarding KCN clinical signs and had normal tomographic studies. **Table 3A** summarizes the individuals by laterality and **Table 3B** represents prevalence by age categories and sex. Unilateral cases were defined as such because the fellow eye did not undergo cataract extraction or were discarded by the methods described herein. The prevalence of SKCN did not increase with increasing age for the cohort (p = 0.11, logistic regression), but SKCN was significantly higher among females aged 60-69 (p < 0.001) and 80-89 years compared to their male counterparts (p < 0.001). Due to limitations of statistical power, comparison by race required dichotomization into white (2709 individuals) and non-white (1119 individuals), in which SKCN was more prevalent among non-whites 34.3% (95%CI, 31.6 – 37.2) compared to whites 20.7% (95%CI, 19.2 – 22.3, p < 0.001).

**Table 3A:** Prevalence of subclinical keratoconus (SKCN) and manifest keratoconus (KCN) by laterality (N = 3828 individuals).

	Frequency	Prevalence
Normal	2796	
SKCN Unilateral Bilateral	517 428	24.7% (95%CI, 23.4 – 26.1)
KCN Unilateral Bilateral	64 23	1.9% (95% CI, 1.6 – 2.4)

**Table 3B:** Prevalence of subclinical keratoconus (SKCN) by age decade and sex (N = 3828 individuals).

	Male (row %)	Female (row %)	Test of proportions (*P* value)
Age 50 – 59 years (n = 341 individuals)	19.7%	22.7%	0.186
Age 60 – 69 years (n = 1108 individuals)	19.2%	26.6%	<0.001
Age 70 – 79 years (n = 1722 individuals)	25.4%	25.5%	0.924
Age 80 – 89 years (n = 623 individuals)	22.3%	31.9%	<0.001
Age ≥90 years (n = 34 individuals)	15.4%	23.8%	0.250
Total individuals by sex	1708	2120	

### 3.3 Tomographic parameters

**Figure 2:**
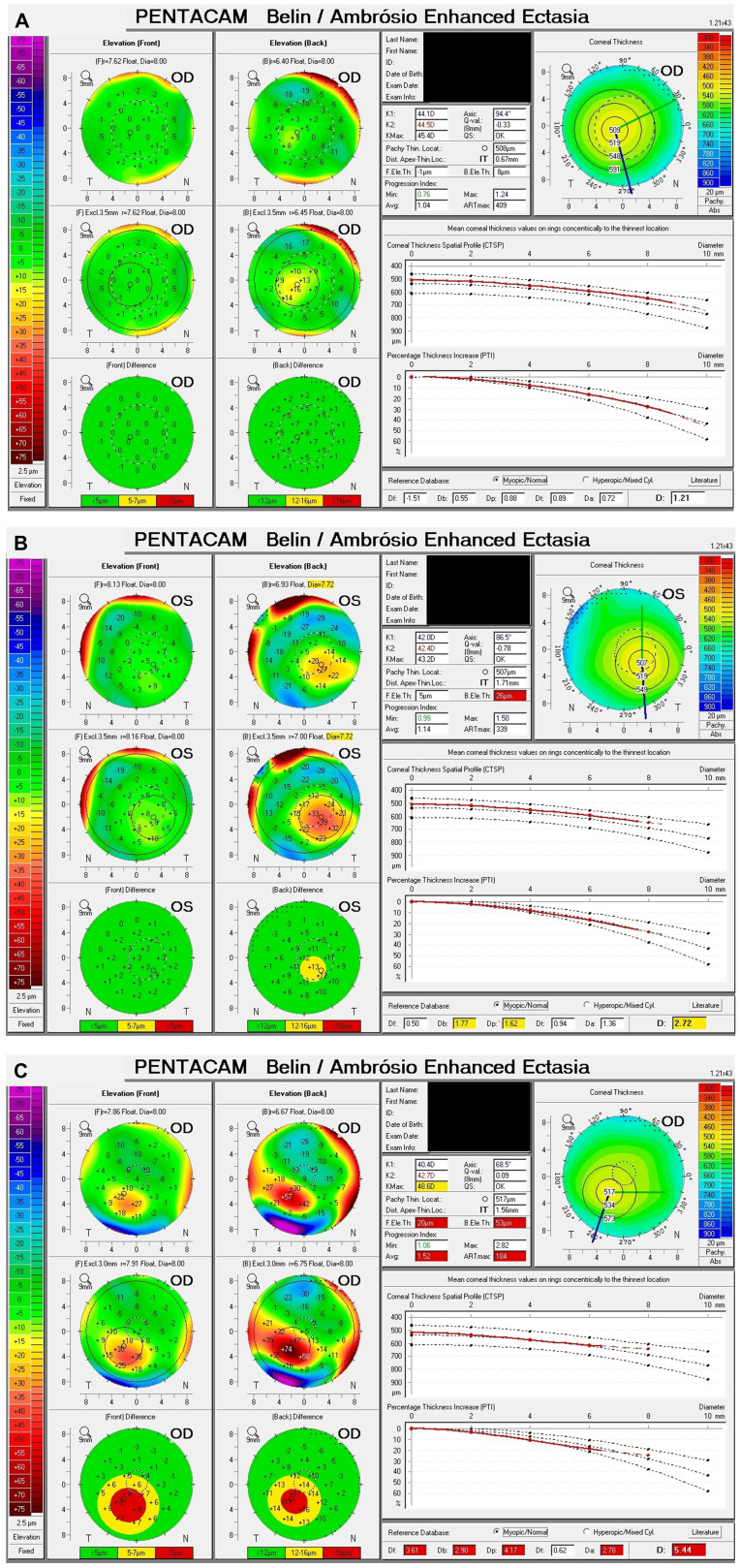
Example Belin/Ambrósio displays of normal, subclinical keratoconus (SKCN) and keratoconus (KCN). **(A)** Normal: BAD-D 1.21, back elevation thinnest point 8 μm. **(B)** SKCN: BAD-D 2.72, back elevation thinnest point 26 μm (highly deviated), index of vertical asymmetry 0.33 (highly deviated), keratoconus index 1.09 (highly deviated). **(C)** KCN: BAD-D 5.44, back elevation thinnest point 53 μm (highly deviated), pachymetric progression index average 1.52 (highly deviated), Ambrósio relational thickness max 184 (highly deviated).

Tomographic parameters are displayed in **Table 4**. The mean BAD-D for the SKCN group was 2.00 ± 0.67 (95%CI, 1.97 – 2.03) versus 0.86 ± 0.66 (95%CI, 0.84 – 0.87) for the normal group (p < 0.001). As a comparison, the mean BAD-D for the KCN group was 6.32 ± 3.62 (95%CI, 5.65 – 6.99). There was a significant difference between the normal and SKCN groups for all key Pentacam-derived curvature-based, elevation, pachymetric, aberrometric, and BAD-D regression parameters. Representative examples of the BAD-D displays for normal, SKCN and KCN eyes are provided in **Figure 2**.

**Table 4:** Tomographic parameters among normal, subclinical keratoconus (SKCN) and manifest keratoconus (KCN) [N = 5592 eyes].

Values provided are mean ± standard deviation (95% confidence interval)	Normal (n = 4109)	SKCN (n = 1373)	KCN (n = 110)	Difference between normal and SKCN (*P* value)
K_max_ Front (Diopters)	45.10 ± 1.70 (45.05 – 45.15)	46.06 ± 1.77 (45.97 – 46.14)	50.15 ± 5.02 (49.22 – 51.08)	<0.001
Anterior corneal astigmatism (Diopters)	1.05 ± 0.95 (1.02 – 1.08)	1.17 ± 1.12 (1.11 – 1.23)	3.49 ± 2.55 (3.01 – 3.96)	0.0004
Index of height asymmetry (IHA)	5.83 ± 4.95 (5.69 – 5.07)	6.93 ± 6.18 (6.63 – 7.24)	16.69 ± 17.64 (13.42 – 19.96)	<0.001
Index of height decentration (IHD)	0.013 ± 0.008 (0.012 – 0.013)	0.016 ± 0.011 (0.015 – 0.017)	0.064 ± 0.054 (0.054 – 0.074)	<0.001
Index of surface variation (ISV)	18.40 ± 3.06 (18.17 – 18.63)	22.37 ± 10.84 (21.84 – 22.91)	52.84 ± 29.80 (47.31 – 58.37)	<0.001
Index of vertical asymmetry (IVA)	0.146 ± 0.072 (0.143 – 0.148)	0.184 ± 0.098 (0.179 – 0.188)	0.518 ± 0.355 (0.452 – 0.584)	<0.001
Keratoconus index (KI)	1.023 ± 0.0303 (1.022 – 1.023)	1.030 ± 0.0404 (1.027 – 1.031)	1.11 ± 0.107 (1.092 – 1.132)	<0.001
Anterior radius of curvature in 3.0mm zone (ARC, in mm)	7.74 ± 0.26 (7.74 – 7.75)	7.62 ± 0.29 (7.61 – 7.64)	7.30 ± 0.81 (7.15 – 7.45)	<0.001
Posterior radius of curvature in 3.0mm zone (PRC, in mm)	6.31 ± 0.24 (6.30 – 6.32)	6.11 ± 0.23 (6.096 – 6.12)	5.48 ± 0.67 (5.36 – 5.61)	<0.001
Thinnest pachymetry (μm)	546.8 ± 31.9 (545.8 – 547.7)	521.9 ± 31.8 (520.4 – 523.6)	480.6 ± 53.4 (470.6 – 490.5)	<0.001
Ambrósio relational thickness average (ARTavg)	623.6 ± 119.8 (620.2 – 627.1)	484.2 ± 80.9 (480.2 – 488.2)	278.2 ± 119.6 (256.0 – 300.4)	<0.001
Ambrósio relational thickness max (ARTmax)	477.9 ± 93.3 (475.2 – 480.5)	365.0 ± 65.0 (361.8 – 368.2)	192.6 ± 85.2 (176.8 – 208.4)	<0.001
BAD-D (final D)	0.86 ± 0.66 (0.84 – 0.87)	2.00 ± 0.67 (1.97 – 2.03)	6.32 ± 3.62 (5.65 – 6.99)	<0.001
BAD-Df	0.24 ± 1.22 (0.21 – 0.27)	0.54 ± 1.70 (0.46 – 0.63)	5.53 ± 6.52 (4.32 – 6.74)	<0.001
BAD-Db	0.52 ± 0.99 (0.49 – 0.55)	1.31 ± 1.20 (1.25 – 1.37)	5.88 ± 5.58 (4.85 – 6.92)	<0.001
BAD-Dp	-0.019 ± 1.11 (-0.051 – 0.012)	1.30 ± 1.34 (1.23 – 1.37)	7.06 ± 5.71 (6.00 – 8.12)	<0.001
BAD-Dt	-0.20 ± 0.89 (-0.22 – -0.17)	0.56 ± 1.23 (0.50 – 0.62)	2.03 ± 1.97 (1.66 – 2.39)	<0.001
BAD-Da	0.092 ± 0.85 (0.068 – 0.12)	1.12 ± 0.59 (1.095 – 1.15)	2.70 ± 0.79 (2.55 – 2.84)	<0.001
BAD-Dy	0.65 ± 1.28 (0.62 – 0.69)	1.16 ± 1.34 (1.097 – 1.23)	1.82 ± 2.36 (1.39 – 2.26)	<0.001
HOA front (root mean square sum)	0.57 ± 0.22 (0.56 – 0.58)	0.66 ± 0.31 (0.64 – 0.68)	1.68 ± 1.12 (1.47 – 1.89)	<0.001
Z_3_^-1^ front (vertical coma)	-0.18 ± 0.20 (-0.19 – -0.17)	-0.23 ± 0.24 (-0.25 – -0.22)	-1.11 ± 1.071 (-1.32 – -0.91)	<0.001
Z_5_^-1^ front (secondary vertical coma)	-0.023 ± 0.061 (-0.025 – -0.021)	0.038 ± 0.089 (0.033 – 0.043)	0.15 ± 0.13 (0.13 – 0.18)	<0.001
Z_3_^-1^ back (vertical coma)	0.010 ± 0.067 (0.0085 – 0.013)	0.035 ± 0.078 (0.031 – 0.039)	0.28 ± 0.32 (0.22 – 0.34)	<0.001

BAD-D, Belin/Ambrósio final index; BAD-Df, deviation in difference map of corneal front surface; BAD-Db, deviation in difference map of corneal back surface; BAD-Dp, deviation of the averaged pachymetric progression; BAD-Dt, deviation of the corneal thickness at thinnest point; BAD-Da, deviation of Ambrósio Relational Thickness and the final D; BAD-Dy, deviation of the thinnest point on y axis; CI, confidence interval; HOA, higher order aberrations.

### 3.4 Visual and refractive outcomes

For consistency and maximizing internal validity, the outcomes data were analyzed for a single senior surgeon (SG) and his routine cataract surgery cases within a one year period, July 2021 to June 2022. Cases falling within three weeks on either side of the time cutoffs were included for analysis. A total of 606 eyes were analyzed, and the outcomes are summarized in **Table 5**. The prevalence proportion of SKCN in this sub-group of 606 eyes (23.1%) was not significantly different compared to the proportion for all 5592 eyes (24.5%, p = 0.429). In a hierarchical logistic regression model, in which an eye was set as unit of analysis and the individual set as a cluster/link variable, the odds for improvement in VA at one month were no different between normal versus SKCN eyes (p = 0.793) and normal versus KCN eyes (p = 0.707). The proportion of eyes with hyperopic outcome was 5.3% for normal eyes, 2.8% for SKCN eyes, and 0% for KCN; the odds of a hyperopic outcome were no different comparing the groups. There was also no difference in odds between normal and SKCN eyes in regard to a refractive outcome within ±0.5 diopter (p = 0.904) and ±1.0 diopter (p = 0.370) of the intended refractive target. However compared with normal eyes, KCN eyes were less likely to achieve refraction within ±0.5 diopter (odds ratio 0.016 [95%CI 0.0031 – 0.079], p < 0.001) of the target and even less likely within ±1.0 diopter (odds ratio 0.0053 [95%CI 0.00084 – 0.033], p < 0.001) of the target. Older age was associated with a higher likelihood albeit small magnitude of achieving the refractive target among normal, SKCN, and KCN eyes: within ±0.5 diopter (odds ratio 1.02 per year of age [95%CI 1.01 – 1.03], p < 0.001) and within ±1.0 diopter (odds ratio 1.02 per year of age [95%CI 1.01 – 1.03], p = 0.001). Among all three groups, the use of a multifocal or extended depth of focus (EDOF) IOL did not diminish the odds of achieving a refractive outcome within ±0.5 diopter (p = 0.774) and within ±1.0 diopter (p = 0.258). In a hierarchical linear regression model, SKCN increased the post-operative month one manifest refraction cylinder by a mean of 0.17 diopter (95%CI, 0.058 – 0.28, p = 0.003), and KCN increased the cylinder by a mean of 0.43 diopter (95%CI, 0.037 – 0.83, p 0.032). The use of a toric IOL significantly decreased the cylinder by a mean of -0.20 diopter (95%CI, -0.32 – -0.086, p = 0.001), and astigmatic incisions significantly decreased the cylinder by a mean of -0.18 diopter (95%CI, -0.27 – -0.080, p < 0.001).

**Table 5:** Visual and refractive outcomes of a one-year cohort performed by a single cataract surgeon July 2021 to June 2022 (N = 606 eyes).

	Normal (n = 452)	SKCN (n = 140)	KCN (n = 14)	Difference between normal and SKCN (*P* value)
BAD-D: mean ± standard deviation	0.84 ± 0.56	2.03 ± 0.71	7.54 ± 2.66	<0.001
Age: mean ± standard deviation	72.9 ± 7.9	73.7 ± 8.4	67.7 ± 8.6	0.368
Female: n (%)	239 (52.8)	87 (62.1)	10 (71.4)	0.051
Baseline DCVA, logMAR: median (range)	0.176 (0 – 0.602)	0.176 (0 – 0.510)	0.301 (0 – 0.477)	0.949
Refractive target, sphere: median (range)	0.0 (-2.5 – 0.0)	-0.25 (-2.5 – 0.0)	-0.75 (-2.5 – 0.0)	0.373
IOL implanted: n (%)				
J&J Eyhance	228 (50.4)	63 (45.0)	3 (21.4)	0.302
J&J Eyhance toric	78 (17.3)	23 (16.4)	6 (42.9)	
J&J Tecnis monofocal	53 (11.7)	26 (18.6)	1 (7.1)	
J&J Symfony	23 (5.1)	6 (4.3)	4 (28.6)	
Zeiss CT Lucia 602	20 (4.4)	3 (2.1)		
J&J Synergy	18 (4.0)	4 (2.9)		
B&L Envista	11 (2.4)	8 (5.7)		
J&J Symfony toric	9 (2.0)	2 (1.4)		
RxSight Light Adjustable	7 (1.6)	4 (2.9)		
J&J Synergy toric	3 (0.7)	1 (0.7)		
J&J Tecnis monofocal toric	2 (0.4)	0 (0.0)		
Intraoperative astigmatic incisions	184 (40.7)	51 (36.4)	1 (7.1)	0.366
POM1 DCVA, logMAR: median (range)	0 (0 – 0.301)	0 (0 – 0.301)	0.048 (0 – 0.398)	0.175
Change in DCVA: baseline to POM1				
Improvement: n (%)	367 (81.2)	114 (81.4)	10 (71.4)	0.752
Same: n (%)	80 (17.7)	23 (16.4)	2 (14.3)	
Worse: n (%)	5 (1.1)	3 (2.1)	2 (14.3)	
POM1 MRx, SE: mean ± standard deviation	-0.56 ± 0.85	-0.78 ± 0.92	-0.87 ± 1.034	0.0078
POM1 MRx, cylinder: mean ± standard deviation	0.51 ± 0.47	0.68 ± 0.69	0.93 ± 0.43	0.0058
POM1 MRx within ± 0.5 diopter of target: n (%)	427 (94.5)	133 (95.0)	4 (28.6)	0.904
POM1 MRx within ± 1.0 diopter of target: n (%)	441 (97.6)	135 (96.4)	4 (28.6)	0.462
POM1 MRx hyperopic surprise: n (%)	24 (5.3)	4 (2.8)	0 (0.0)	0.249

BAD-D, Belin/Ambrósio final index; B&L, Bausch & Lomb; DCVA, distance corrected visual acuity; IOL, intraocular lens; J&J, Johnson & Johnson Vision; KCN, manifest keratoconus; MRx, manifest refraction; POM1, post-operative month one; SE, spherical equivalent; SKCN, subclinical keratoconus.

‡Zeiss CT Lucia IOL was intended and this IOL was implanted during primary cataract extraction surgery not as secondary IOL surgery.

## 4 Discussions

Based on a combination of tomographic parameters previously validated in the literature, we have determined that one in four individuals undergoing routine, age-related cataract extraction at a tertiary referral center have SKCN. We arrived at this estimate using a BAD-D cutoff ≥1.7 with an abnormally high value for at least one of seven additional variables including posterior elevation at thinnest point, ISV, IVA, HOA front corneal surface, vertical coma of the front and back surface and secondary vertical coma of the front surface to define SKCN. The BAD-D cutoff used in this study is both higher than the threshold previously determined in the literature (13, 15, 20–25) and the threshold recommended by the manufacturer of 1.6 (16). Despite this, eyes with SKCN in our cohort had a mean BAD-D of 2.00 with a lower limit of the 95% confidence interval at 1.97; therefore, these SKCN eyes were deviated at least two standard deviations from age-matched normals. While the 24.7% prevalence from our study seems like a high number, the prevalence proportion for KCN of 1.9% is well within range of prevalence proportions from other contemporaneous clinical cohorts and modern population studies (e.g., 2.3 – 8.9%) (2, 17, 18, 26–29). Conventionally, SKCN is on the spectrum with KCN especially in adolescents, but clinically in this older age group, ectatic changes have likely stabilized. Therefore, we surmise SKCN at this age group is a fairly benign entity, and our visual and refractive outcomes data support this notion.

Because there is no consensus definition of SKCN, we used a two-stage process reliant on tomographic parameters to screen-in cases. The BAD-D regression model accounts for nine parameters evaluating corneal anterior surface, posterior surface, and pachymetry as described by Belin and Ambrósio (16). Comparing an individual’s tomographic data to age-matched normals, the BAD-D represents the standard deviation from the normal in which 1.6 or higher is considered the cutoff for a KCN suspect, which is of greater significance than SKCN (30, 31). At 1.65, the false positive rate for even clinically manifest KCN is only 5% (32). Its validity as a screening tool has achieved widespread consensus (13, 15, 24, 25, 33–36), with area under the receiver operating characteristics curve (AUROC) for KCN ranging from 0.83-0.93 (13, 15, 21, 31, 33–35). Even among SKCN and normal eyes, the BAD-D is replicable with low variations on repeat scans within a similar session (37). Using a similar method to ours, the BAD-D was used solely in determining the prevalence of KCN of 1.2% in Western Australia (38). Other parameters for early detection have been validated alongside the BAD-D. Luz and colleagues developed a regression model that also includes the Ambrósio relational thickness max (ARTmax), enhanced best fit sphere front (BFS front), elevation back at thinnest point and within the central 4mm zone, and max pachymetry. Awad and colleagues found ARTmax to be more sensitive and specific than the BAD-D in differentiating KCN suspect from normal. Hashemi and colleagues added ISV and IVA (21). Heidari and colleagues determined root mean square sum of HOAs of the front surface, 3^rd^ order vertical coma of the front and back surfaces, and 5^th^ order vertical coma of the front surface were most important parameters in distinguishing KCN and normal eyes (5). Consistent with the literature, we found significant differences between normal and SKCN in regard to ARTmax, max pachymetry, ISV, IVA, and the aforementioned aberrometric parameters.

Indeed, the Pentacam parameters were crucial for screening-in eyes suggestive of SKCN. To avoid inaccurate application of these parameters, we excluded cases of poorly controlled ocular surface disease as this usually led to high values for ISV and BAD-Df, despite within normal range values for the other BAD-D indices, IVA, KI, and ART. For a similar reason, cases with contact lens warpage were excluded from analysis. Indeed, the corneal epithelium’s effect on total corneal refractive power can affect the normal air-epithelial interface and lead to false diagnosis of SKCN due to surface irregularity (39). In cases with previous keratorefractive surgery, including photorefractive keratectomy (PRK) and laser *in-situ* keratomileusis (LASIK), these procedures resulted in highly deviated ISV, IVA, BAD-Df and BAD-Dp. The assumptions of the enhanced best fit sphere cannot hold, and these eyes were excluded from analysis. While it is possible these post-refractive eyes could have SKCN, the signal-to-noise ratio was far too low to consider these eyes for inclusion, and post-keratorefractive ectasia and cataract surgery is beyond the purview of this study.

Since SKCN and KCN are on a spectrum, we acquired medical and other ocular history to assess for associations with SKCN. Atopy has long been associated with KCN due to its potentiation of eye rubbing (14), but there was no significant difference in atopy among normal, SKCN, and KCN groups in our cohort. Similarly, allergic conjunctivitis was similarly prevalent among all three groups in our cohort. Hashemi and colleagues’ systematic review showed allergy, asthma, eczema were associated with KCN, while diabetes mellitus type I and II were not (1). In diabetes mellitus, advanced glycation end product-mediated collagen crosslinking helps stabilize the cornea and may prevent progression of KCN (40), which could be why we found a higher proportion of diabetics in the SKCN group compared to KCN. Tobacco smoke has been associated with toxic-mediated corneal crosslinking (41) with increased biomechanical rigidity, and we found far more ex-smokers and current smokers in the SKCN group than KCN. The deleterious health effects of smoking heavily outweigh any consideration in counseling patients, but the patterns from our cohort do reflect known associations in the literature and reinforce the notion that SKCN and KCN are on a spectrum. The prevalence of diagnosed obstructive sleep apnea (OSA) was similar across all three groups in our cohort, though there was likely a higher prevalence of undiagnosed OSA and associated floppy eyelid syndrome, in which patients with KCN are 1.8 times more likely to have OSA despite conflicting studies in the literature (42). Of note, there was a significantly higher proportion of obese individuals in the KCN group in our cohort reflecting likely underdiagnosed OSA. In this age group of individuals 50 years and older, we are not seeing the same magnitude of impact by habit (e.g., eye rubbing) and environment since these individuals’ SKCN course must have stabilized. Indeed, in the only population study that assesses KCN across the lifespan from South Korea, these risk factors become diluted with aging. The authors found allergic conjunctivitis was only slightly more prevalent among those with KCN (35.5%) versus normal (31.0%) and atopy, asthma, connective tissue disorders, and sleep apnea were no more prevalent among those with KCN than normal eyes (43). Our cohort was consistent with this South Korea-based study. Finally, it has been documented that Asian and Arab ethnicities have a higher prevalence of KCN (44); our cohort showed a higher prevalence of SKCN and KCN among non-whites.

Since the biomechanical properties of corneas in SKCN have likely stabilized by the time an individual reaches cataract extraction age, we hypothesize the impact on visual and refractive outcomes is likely negligible as a best-case scenario and is at most, a factor to consider in pre-operative counseling to modulate expectations. This hypothesis is supported by our results showing older age individuals were more likely to be within ±0.5 and ±1.0 diopter of the refractive target. Kamiya and colleagues showed in mild stage KCN eyes undergoing cataract extraction and IOL implantation, 80% of eyes refracted within ± 1.0 diopter by spherical equivalent of the intended target with an average of 0.52 diopter more hyperopic than predicted by a combination of third and fourth generation IOL formulas (45). This proportion of refractive outcome in KCN eyes was far lower in our cohort at 28.6% due to the high residual astigmatism. Nevertheless compared to normal eyes, SKCN eyes had a near equivalent proportion of eyes with improvement in VA and reached a similar median post-operative month one VA. The proportions of SKCN eyes refracting within ±0.5 and ±1.0 diopter of the refractive target were high, 95.0% and 96.4%, respectively, and were nearly equivalent to the proportions in normal eyes. The median refractive targets of SKCN and KCN were more myopic which may account for the lower proportions of hyperopic surprises in SKCN and KCN eyes compared to normal eyes. Our practice favors use of monofocal IOLs in eyes with KCN, and currently this is the prevailing practice pattern globally (46, 47). The use of toric IOLs and astigmatic incisions, the vast majority placed by a femtosecond laser, were beneficial in reducing residual astigmatism in SKCN eyes. We did not use KCN-specific IOL formulas in IOL selection, but these formulas likely have a beneficial role and ought to be considered as additional data point for SKCN given their exceptional performance compared to other fourth generation non-KCN specific formulas (48).

Our study is limited by its retrospective design and therefore we could not assess through formal questionnaire behavioral factors, such as eye rubbing, and subclinical medical morbidities such as snoring for OSA. Another major limitation is the patient population drawn from a single university-based tertiary referral center, in which individuals typically have higher acuity ocular disease than in the average population of the United States seeking cataract surgery. A few mitigating factors include acquisition of Pentacam scans by a core cadre of experienced technicians specializing in cataract pre-operative imaging, a consistent pattern of practice in pre-operative evaluation, dry eye management, and astigmatism management by four cataract surgeons (eg. toric marking in upright position or loading biometry data into the femtosecond laser); with regard to the visual and refractive outcomes, there was a consistent pattern of IOL selection and surgical management by a single cataract surgeon in selecting a one-year cohort. We did not formally assess eye rubbing behavior as this was not part of the routine cataract surgery intake questionnaire; thus, the potential association of more eye rubbing with chronic conditions such as DES, allergic conjunctivitis, or medicamentosa from glaucoma eye drops was not factored into the tomographic results and the prevalence of ocular and systemic conditions. Dynamic Scheimpflug analysis has become a powerful tool owing to its appreciation of the biomechanical properties of the cornea and necessarily improves accuracy of the diagnostic yield (25, 49); however, an applanation device coupled to a Scheimpflug camera, such as a Corvis ST (Oculus GmbH, Wetzlar, Germany), was not available and has not been routinely applied in the setting of cataract surgery preoperative evaluation, even for known manifest KCN. Anterior segment optical coherence tomography is another modality that, while not implemented in our cohort, may have a role in increasing sensitivity of case detection of SKCN in future clinical practice (50). In the outcomes analysis, there was a bias toward Johnson & Johnson Vision family of IOLs, and our conclusions should cautiously be extended to other IOL manufacturers. Secondly, other characterizations of visual function such as contrast sensitivity and visual quality of life (NEI VFQ-25) were not acquired. Nevertheless, our study has a large sample size powered to answer the primary question on prevalence in this specific demographic and is the only known study among the ageing population undergoing cataract extraction.

## 5 Conclusion

Our prevalence figure of one in four patients should be interpreted with caution such that SKCN should be an entity to screen for in cataract pre-operative evaluation, but we are not advocating for definitive changes in standard practice since the major indicators for cataract surgery outcomes are comparable to normal eyes. The methods herein depend on a combination of previously validated parameters and can lend itself to a considerable number of false positives, which we have spent considerable effort controlling for and ruling out such false positives. Uncovering SKCN does not absolutely preclude use of toric, EDOF, or multifocal IOLs. On the contrary, toric IOLs and astigmatic incisions aid in astigmatism management. At the very least, if a patient without identifiable contributing factors to KCN and the eye has a BAD-D ≥1.7, the clinician can use this information to better modulate patient expectations.

## Data availability statement

The raw data supporting the conclusions of this article will be made available by the authors, without undue reservation.

## Ethics statement

The studies involving humans were approved by University of California Irvine. The studies were conducted in accordance with the local legislation and institutional requirements. The ethics committee/institutional review board waived the requirement of written informed consent for participation from the participants or the participants’ legal guardians/next of kin because Retrospective database study in which acquiring consent from each patient would prohibit execution of this type of study, which is done routinely in modern medical research.

## Author contributions

TT: Conceptualization, Data curation, Formal analysis, Investigation, Methodology, Project administration, Software, Supervision, Validation, Visualization, Writing – original draft, Writing – review & editing. AM: Conceptualization, Formal analysis, Methodology, Writing – original draft, Writing – review & editing. VN: Data curation, Methodology, Writing – original draft, Writing – review & editing. PC: Conceptualization, Writing – review & editing. MW: Conceptualization, Investigation, Methodology, Supervision, Writing – review & editing. SG: Conceptualization, Data curation, Formal analysis, Funding acquisition, Investigation, Methodology, Project administration, Resources, Supervision, Writing – original draft, Writing – review & editing.
